# Personalisation schemes in social care and inequality: review of the evidence and early theorising

**DOI:** 10.1186/s12939-019-1075-2

**Published:** 2019-11-06

**Authors:** Gemma Carey, Brad Crammond, Eleanor Malbon

**Affiliations:** 1 Centre for Social Impact, University of New South Wales, UNSW, Sydney, NSW 2052 Australia; 2 Monash University, Melbourne, Australia

**Keywords:** Personalisation, Inequality, Individual-budgets, Bourdieu, Social class, Ex-developed countries

## Abstract

**Background:**

Personalisation is a growing international policy paradigm that aims to create both improved outcomes for individuals, and reduce fiscal pressures on government, by giving greater choice and control to citizens accessing social services. In personalisation schemes, individuals purchase services from a ‘service market’ using individual budgets or vouchers given to them by governments. Personalisation schemes have grown in areas such as disability and aged care across Europe, the UK and Australia.

There is a wealth of evidence in public health and health care that demonstrates that practically all forms of social services, programs and interventions produce unequal benefit depending on socio-economic position. Research has found that skills required to successfully negotiate service systems leads to disproportionate benefit to the ‘middle class. With an unprecedented emphasis on individual skills, personalisation has even greater potential to widen and entrench social inequalities. Despite the increase in numbers of people now accessing services through such schemes, there has been no examination of how different social groups benefit from these schemes, how this widens and entrenches social inequities, and – in turn – what can be done to mitigate this.

**Methods:**

This article presents a meta-review of the evidence on personalisation and inequality. A qualitative meta-analysis was undertaking of existing research into personalisation schemes in social services to identify whether and how such schemes are impacting different socio-economic groups.

**Results:**

No research was identified which seeks to understand the impact of personalisation schemes on inequality. However, a number of ‘proxies’ for social class were identified, such as education, income, and employment, which had a bearing on outcome. We provide a theoretical framework for understanding why this is occurring, using concepts drawn from Bourdieu.

**Conclusion:**

Personalisation schemes are likely to be entrenching, and potentially expanding, social inequalities. More attention needs to be given to this aspect of personal budgets by policymakers and researchers.

## Background

There has been interest from a range of disciplines regarding the way the middle class use welfare services and the disproportionate benefit they derive from them [1–6]. In health, this phenomena became known as the inverse care law [4], a trend that has subsequently been found in the field of health promotion and dubbed the ‘inverse prevention law’ [7]. Public health has also put considerable energy into documenting ‘social gradients’ (whereby lower socio-economic groups experience poorer health and wellbeing than higher socio-economic groups, following a ‘gradient’) [8]. In social policy the phenomena has been most studied in the area of education services in industrialised countries [6], which follow the same pattern. Concern for how the middle class use and benefit from social services sits within a broader and long running frame of how effective welfare states are at redistributing social benefit to the population [9–11].

While interest in the effects of welfare states on inequality are long standing, welfare states in industrial countries have gone through major transitions over the past three-to-four decades. Scholars in the field have roughly grouped these into three phases: public administration, new public management and new public governance [12]. While these phases over-lap they can be characterised as a gradual shift towards externalisation of public services. This has been driven by both supply and demand side arguments. On the supply side, the emergence of new public management saw governments argue that services are more efficiently and effectively delivered by non-state actors [12, 13]. With regards to demand, third sector and other non-government organisations were believed to be able to better meet the varied needs of citizens [14]. While in practice we still see facets of new public management in play, Osborne [12] contends that we have entered an era of new public governance. New public governance is characterised by complexity and plurality – new practices are being layered on top of old, with largely unknown effects [12, 15]. Of particular relevance to those concerned with the distributional effects of welfare states is the emergence of personalisation and/or individualisation agendas under new public governance.

Personalisation is characterised by a range of different mechanisms and administrative structures, however at the core of this paradigm is a concern for greater choice and control for public service users through personal budgets or voucher systems [16, 17]. Here, citizens ‘purchase’ services that best meet their needs. While the concept is relatively simple, the administrative structures through which such schemes are administered are hugely complex [18]. They require considerable skill on behalf of citizens to navigate [18–22]. Despite the growth of personalisation schemes internationally [23], there has been no examination of whether these schemes follow inverse care principles.

Matthews and Hastings [5, 6] have argued that the middle class derive greater benefit from welfare services because of an alignment between their ‘habitus’ (a concept drawn from the work of Bourdieu [24, 25] and welfare services. That is, welfare services are calibrated, so to speak, to a middle class ‘doxa’, which can broadly be understood as sets of norms and values [25]. Additionally, the middle class have skills and knowledge which enable them to better negotiate administrative systems and self-advocate [5, 6]. Given the individualisation of social services under personal budgets, we might hypothesise that they are more likely to result in disproportionate benefits to the middle class.

In this paper we provide a systematic review of the evidence of personalisation schemes and their likely effects on inequality. We found that no empirical studies have been conducted that seek to analyse whether a social gradient exists in personalisation, with those in higher socio-economic groups drawing more benefit (social or health) from such schemes than lower socioeconomic groups. However, our review of the evidence uncovered a key set of capabilities required to successfully navigate personalisation schemes and individual budgets, which can loosely be considered proxies for being middle class (i.e. they are found more commonly amongst the middle class). Using previous conceptual work on why the middle class disproportionately benefit from welfare services [5, 6], we theorise how and why this may be occurring in personalisation schemes. We argue that there is a strong likelihood that personalisation schemes are increasing and/or entrenching inequalities.

Firstly, we provide an overview of the personalisation agenda as an international trend in social care, before describing the methods of the study. We then present the findings, and draw on these in combination with the work of Bourdieu to create our conceptual framework.

### The personalisation agenda

Since the 1980s governments have sought to give citizens greater choice and control of the public services they use [14]. This has resulted in the emergence of various types of public sector markets created through contracting and tendering of non-government organisations who, it is argued, are more responsive to community needs than the ‘one size fits all’ approach of government [26, 27]. In the mid 1990s new mechanisms emerged to give choice and control to citizens, sometimes referred to as ‘particularist’ approaches [28, 29]. Particularism aims to address differences between individuals on the basis of diversity of needs, moral frameworks and social expectations [30]. Critically, particularist approaches are said to allow for, and encourage, empowerment [31]. One of the most prominent examples of the particularist paradigm is the growing personalization agenda and the use of individual budgets for citizens.

At its best, particularism should see different social “groups assert [ing] particular welfare needs on the basis of empowered identities” [31] (p 332). At its worst, critics suggest that particularism’s emphasis on choice and pluralism risks subverting efforts to combat inequality by eroding the collective approach to the welfare state [29, 32]. Particularist approaches, and personalization, are premised on the notion that individual citizens know what their service needs are and are able to clearly articulate these to state officials (who issue individual budgets), and negotiate care markets in order to purchase required services. Hence, personalisation schemes put unprecedented emphasis on individuals to navigate services and advocate for their rights [22].

Personalisation first emerged in the United Kingdom in adult social care, inspired in part by earlier social movements in the US [33]. This was part of both a fight for redistribution and recognition by disability advocates [34]. On the supply side, personalisation and particularist approaches have been said to be more economically efficient [35]. Hence, the personalisation agenda has also emerged from broader pressures on welfare states. Faced with a range of fiscal and social pressures, we have seen shifts in many industrialised countries away from collective social welfare provision in favour of markets and ‘self-directed care’ [35]. To date, personalisation has emerged in the UK, Germany, Scandinavia and the Netherlands and more recently Australia, in areas such as aged care, disability and health [36]. The common principles of personalisation schemes worldwide are that:

- Participants are conceptualised as consumer-agents in care services, able to exercise enhanced choice over how their needs should be met and thus increasing their experience of control
- In the majority, personalisation schemes utilise individual budgets as a tool to provide choice and control to participants

Whether such approaches create improvements in people’s lives is still a matter of debate [37, 38]. Critically, there is considerable variation in people’s ability to negotiate the systems through which personalization schemes are administered. Previous research indicates that this often plays out along socioeconomic lines.

As argued by Matthews and Hasting [6], research into the distribution of welfare benefits across socioeconomic groups has tended to focus on top-down mechanisms (i.e. policy design and drivers), rather than a more bottom-up focused approach to understanding how individual citizens (or groups of citizens) are able to negotiate social services and their political activity. In education, where there has been a greater focus on bottom-up approaches to understanding such phenomena, research suggests that the middle class are able to draw disproportionate advantages due to normative forces and individual skills that privilege the middle class [6]. Instead, the dominant research focus has been on the mismatch between services and lower socioeconomic users and/or those with complex needs [39–41]. As Matthews and Hastings have argued, this approach problematizes the working class while ignoring micro-level factors that enable the middle class to draw more benefit from services, thereby creating a social gradient. At present, the existence, or potential for an emergence, of a social gradient in care under personalization schemes has not been discussed. Given the growing push towards personalization approaches it is important to understand how they might benefit some individuals over others – creating or entrenching social disadvantage.

## Methods

The intent of this meta-analysis is to search the empirical literature in order to detect patterns in what is, and is not, effective. While meta-analyses often rely on statistical analysis, we took a thematic approach – synthesizing qualitative insights from empirical case studies on personalization. At present, there is no agreed upon method of qualitative research synthesis, and debate in this area has continued for some time [42, 43]. Overall, thematic approaches to meta-analysis seek to uncover concepts and their meanings from the data (rather than pre-determining them), using interpretive approaches to ground the analysis in that data (i.e. existing studies). Thematic approaches are useful for hypothesis generation and explanation of particular phenomena, though provide less of a picture of the context and quality of the individual studies that comprise the review [42].

A recent systematic review of individual budgets was conducted by Dickinson [18], which identifies empirical studies concerned with the outcomes of personalisation schemes, though the focus was not on inequalities and an analysis of this is not provided. Dickinson [18] identified 28 studies between the years 2009–2017. Given personalisation emerged in the early-to-mid 1990s [44], we extended the review back to 1990 using the same search criteria as Dickinson [18], in order to conduct an analysis of personalisation and inequalities. Both studies are limited to personalisation schemes in industrialised countries.

**Fig. 1:**
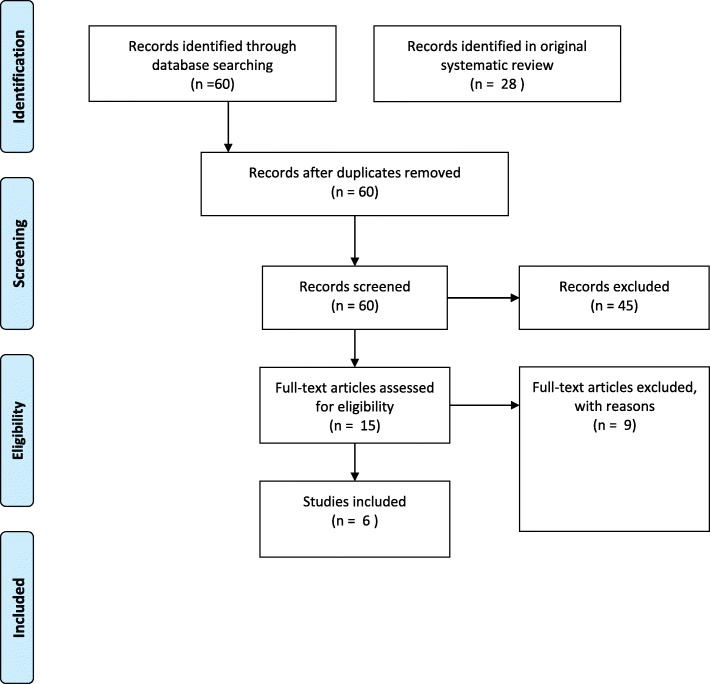
PRISMA Diagram

Searches were conducted in EBSCO, EMBASE, Scopus, Web of Science, Social Services Abstracts and Google Scholar using the following key search terms: ‘personalisation’ AND (‘disability’ OR ‘health’ OR ‘social care’ OR ‘social services), ‘individual funding’, ‘self-directed care’ and ‘market management’ AND (‘disability’ OR ‘health’). The same inclusion and exclusion criteria were applied as the 2017 review. Inclusion criteria were articles published in English that include original/empirical evidence relating to the use of individual funding schemes and their efficacy and were focused on industrialised countries. Articles that were theoretical or conceptual were excluded. Articles that focused on the implications for professional groups without a broader consideration for efficacy or impact of individual budgets and personalisation schemes were also excluded. A further 6 studies were identified from the second review process, and another report was identified through reviewing the citations of studies collected, bringing the total sample to 34 (see Fig. 1).

The full text of 34 articles were coded thematically in order to identify studies that either: (a) were explicitly concerned with the impact of individual budgets and/or personalisation schemes on inequality or (b) described characteristics of individuals associated with good outcomes, such as improved outcomes or satisfaction. These characteristics were recorded. No studies were found for group (a), while 8 studies were found that identified individual characteristics of users of personal budgets that were associated with good outcomes (group b), and is the focus of the remainder of this paper.

## Results

No studies were identified that explicitly examined the outcomes of individuals participating in personalisation schemes by socioeconomic status. This is a major gap in research and understanding of how personalisation schemes operate and the benefits which may or may not flow from them. Through the review, however, a range of factors were identified that were associated with better outcomes. These were: education, being employed, having capable networks and support, knowledge and skills in navigating complex systems, household income, knowledge of where to access information and the capacity to self-manage individual budgets. Table 1 sets out these factors and the supporting studies. In the remainder of this paper we assess these factors against current theorising regarding how some groups derive better outcomes from social services than others.

**Table 1:** Factors influencing good outcomes under personalisation

Studies and sample sizes/methods	Factors identified
Arksey and Baxter (2012) 30 qualitative interviews of service suers	Informal networks and support
	Knowledge and skills in navigating complex bureaucratic systems
	Capacity to self-manage funds
Dew et al. (2013) Focus groups with providers (60 participants)	Knowledge of where to access information
Graham (2015) document review, 15 unstructured qualitative interviews	Informal networks and support
Laragy and Ottoman (2011) Participant observation of families, 12 qualitative interviews with family representatives	Informal networks and support
	Knowledge of where to access information
	Capacity to self-manage funds
Mavromaras et al. (2016) surveys with participants and their families (6246), surveys with providers (2672) qualitative interviews with participants and their families (123) qualitative interviews with survey providers (50) qualitative itnerviews with other stakeholders (114) *Comparison group with clients of non-personalised services –* surveys with service recipients and their families (3877),	Education
	Informal networks and support
	Knowledge and skills in navigating complex bureaucratic systems
	Household income
National Audit Office (2013) Surveys service users (completed sample 69,000)	Informal networks and support
	Knowledge and skills in navigating complex bureaucratic systems
Netten et al. (2012) Randomised control trial of service suers. With an overall sample of 1000 service users Interviews, questionnaires, household demographics	Education
	Employed
Stevens and Wilberforce (2008) 130 qualitative interviews with service providers	Knowledge and skills in navigating complex bureaucratic systems
Warr et al.(2017) 42 qualitative interviews with service users	Education
	Knowledge and skills in navigating complex bureaucratic systems

## Findings and discussion

The factors influencing personalisation outcomes presented in Table 1 can be grouped under three headings, closely corresponding to the types of capital as described by Bourdieu in his sociological work [24, 45]. These three types of capital – economic, cultural and social – combine with a fourth – symbolic capital – to make up a modern conception of social class. Here, symbolic capital refers to the resources available to people on the basis of their social networks and position [24, 45]. We use these concepts to organise our analysis of the intersection between personalisation schemes and ‘class’.

Within this framework, in the contemporary industrialised world social class has become a location-specific conglomeration of financial resources, educational achievements, occupation status, political attitudes, media consumption and cultural habits [46, 47]. Those individuals with greater stocks of capital occupy higher class positions whereas those with diminished access to money, education and company are relegated to lower positions.

The relationship between higher levels of capital and the successful utilisation of personalisation services is cemented by the modes of behaviour generated by particular capital positions, what Bourdieu famously termed *habitus* [45]. Habitus refers to the dispositions produced by class positions; the ways of behaving which are socially consistent with one’s class position. In the realm of personalisation, this internalisation of class position is powerful as an explanation for how modes of behaviour can differ systematically by social group in ways that might advantage or disadvantage them in dealings with social services agencies.

### Economic capital

Economic capital is the most straightforward of the types of capital and encompasses financial resources, principally money. Differential access to economic resources must act as a background to any discussion of reliance upon government welfare [48]. For those families with access to resources independent of government programs, reliance upon government payments is reduced, mitigating (or potentially eliminating) the consequences of failing to obtain an appropriate level of government support.

Without undermining the importance of independent economic resources, only limited evidence was found for a link between family income and the ability to draw benefits from personalised care systems. In our review, Warr et al. [22] found that people living on low incomes, who were also elderly or living with a disability, struggled to cope with the requirements of the scheme (particularly the use of online management portals). The study did not, however, isolate income as a factor. It did find in contrast that existing challenges with bureaucratic systems were exacerbated in low income houses – which is consistent with the importance of independent economic capital. It is also worth noting that no research has explicitly sought to establish whether there is a link between family income and outcomes from personalisation schemes. Hence, no evidence was found that a link does not exist.

What is clear from the studies identified in the review, however, is that additional independent resources can also aid in the procuring of government support. For example, in a disability personalisation scheme Mavromaras et al. [49] found that the size of personal budgets increased when paid advocates were used, making the surplus income required to purchase additional services privately a critical determinant of the level of government support. One of the participants in that study had her plan increase from $700 to $32,000 after employing an advocate.

Consistent with middle-class habitus, financial self-management is a central feature of personalisation schemes with personal budgeting treated as a neutral requirement. The quintessential middle-class value is the calculated accumulation of wealth achieved through careful budgeting [50]. An approach to finances which prioritises the future is central to middle-class habitus and is contrasted to the ‘spontaneous materialism of the working classes’ [24] (p180). The working class’s ‘profligacy’ has been lamented as the cause of its poverty for centuries and this same argument reappears commonly today. Yet budgeting for the future and deferring present gratification is only rational given certain underlying circumstances, especially a consistent inflow of resources and the reasonable expectation of the long-term good health required to enjoy future assets. In circumstances of low income and uncertain health it may be sensible to extract as much pleasure from the available resources as quickly as possible [51].

Indeed, the values of the middle-class are treated as being obvious virtues such that self-management of government benefits are promoted as being ‘empowering’. In our review, Warr et al. (2015) found, however, that many participants within a disability scheme were not able to self-manage their funds in the manner expected by the bureaucracy. Many participants had engaged disability services to manage funds on their behalf, highlighting that a policy which might be empowering to some can be starkly disempowering for others.

### Cultural capital

Cultural capital is often reduced to a person’s formal education though it is designed to encompass how ‘cultured’ a person is. Such a judgement can vary on specifics by time and place – smoking tobacco has, for example, been evidence both of distinction and deprivation – but high cultural capital is universally associated with positions of privilege. In other words, though the relationship between privilege and attending the opera is arbitrary, it is both true that the privileged attend the opera and that those who attend the opera are accorded the privilege of being the sort of person who does so. Cultural capital therefore encompasses the direct skills a person develops through formal education along with the manners of behaving as a ‘cultured’ or ‘educated’ person. Both are advantageous in extracting resources from bureaucracies.

Knowing how to access culturally advantageous systems underpins many of the mechanisms by which cultural capital is inherited. Turning again to the education research, navigating college admissions processes, for example, is enormously daunting for those who are the first in their family to attend college and thus impedes access to the most pure institutionalised cultural capital [52]. The knowledge associated with being able to navigate complex bureaucratic systems is a similarly practical example of cultural capital.

Our findings show that bureaucratic systems are constructed in ways that are familiar to members of the middle class with formal education. Studies within our review that utilised service user interviews described this fact obliquely, describing the process of personalisation as being too complicated and requiring too much effort. Arksey looking at cash-for-care schemes in the UK, for example, found that – when personalisation was optional – people were choosing not to switch to direct payments ‘because they expected the paperwork would be too stressful’ [53] (p 153). Similarly, in Australia’s National Disability Insurance Scheme (where personalisation is the only option if you want to access government payments) the IT portal was seen by respondents as a near insurmountable barrier to accessing government support [22]. One respondent had attended a training day and been handed a 37 page step-by-step manual for accessing what were, by law, benefits to which she was entitled to [22].

In another study, Mavromaras et al. [49] report one respondent saying that ‘*often it’s the more articulate, confident people who actually have the confidence to access advocacy … you actually have to be quite empowered to actually go through that process*”. We can read this as a system designed by and for those with the skills imparted by formal education and location in the middle-classes.

Stevens [54] in a quantitative comparison of individual budgets found that self-perceived health among people with learning disabilities was significantly lower in those who had accepted budgets than those who maintained government-controlled plans. We might expect that the effect of a learning disability would exacerbate the high requirements of confidence, complexity and effort. As Warr et al. [22] report, a participant with a learning disorder reported being satisfied with her current situation but had little sense of how it worked: *“I would say [we get] the support we need. I don’t know what I wanted. I didn’t know what was there”.*

Hence, evidence to date suggests that personalisation schemes require high levels of cultural capital usually associated with the middle class. The unequal distribution of this capital is likely to produce corresponding unequal outcomes for service users.

### Social capital

Social capital refers to the resources an individual can draw upon by virtue of their social connections. Strong social capital can smooth over times of hardship and supplement shortcomings in a person’s individual stores of capital. Theoretically strong social capital is always beneficial – as Bourdieu argues ‘social capital is the aggregate of the actual or potential resources which are linked to … membership in a group’ [24]. Empirically however we can distinguish between social capital which is predominantly supportive and that which is ultimately depletive. Strong social ties among disadvantaged groups who must regularly call upon each other in regular times of crisis can reduce health outcomes, whereas ties with privileged groups who irregularly need such assistance improves health. In other words social relationships only constitute social *capital* when they are supportive.

Many of the studies in our review highlight the importance of informal networks and support to managing under personalisation schemes. Laragy et al. [55] notes the importance of the word of mouth (or ‘grapevine’) in accessing information within a disability personalisation scheme:

However, gaining the necessary information proved to be difficult, as one parent using individual funding without a case manager noted, “I would like it to be easier to access information—it takes a lot of time—I start with [government department], I use the [agency], and most importantly I use the grapevine.” (p 23)

Similarly Mavromas [49] quotes a respondent as saying: “… one of the things that the whole plans come down to is advocacy. If you haven’t got a good advocate to make the plans, you’re in trouble.”

Advocacy takes on four forms in this context: self-advocacy, familial advocacy, not-for-profit advocacy and privately paid advocacy [56]. Paid advocacy, being dependent upon financial resources, is subject to all the shortcomings discussed under economic capital. As with the other categories, those best able to perform effective self-advocacy are already those least disadvantaged. Familial ties are in many ways the most fundamental form of social capital. Bestowed at birth according to the social position of the existing members, familial social capital shapes a person’s opportunities and resources throughout life. Indeed familial advocacy exists as a category precisely because of its strength as resources that can be called upon in times of need – such as obtaining an appropriate level of welfare support from government.

Advocacy by not-for-profit organisations on behalf of the disadvantaged is the key example of bridging social capital, where expertise is provided across class groups. However advocacy systems themselves can be complex and in many cases resources are scarce, which means not everyone who needs an advocate gets one. While advocacy boosts the size of budgets, it too is mediated by cultural capital. For example, sourcing and access to advocates or ability to pay them can be dictated by cultural capital. Moreover, advocates themselves may ascribe to middle class ‘doxa’. Some advocates may make decisions based upon genuine need or an effort to ameliorate the unequal class effects of personalisation. However, if they are swayed by the same factors as the bureaucracy itself – such as how ‘politely’ the client behaves – then they will leave the existing unfair structures untouched.

### Symbolic capital

The prestige accorded to some actors within a field is their symbolic capital. It is symbolic in the sense that it is the symbol of one’s position in relation to the other types of capital. In many cases this will be a direct relationship: in many fields a person’s symbolic capital will rise as she accumulates more money, culture and friends. In other cases the relationship will be more complex, such as when cultural elites eschew the pursuit of money or financial elites are hostile to cultural products [45]. In either case symbolic capital is achieved through accumulation of the types of capital valued by occupants in that field.

Ultimately, the effect of symbolic capital is to legitimate the other forms of capital, transforming the unequal distribution of capital into the appearance of dignity and prestige. Each of the examples we have considered so far coalesce as symbolic capital to enhance the position of the middle-class in its dealings with bureaucratic structures.

Advocates, however, can play a powerful role in redistributing this symbolic capital. By taking on cases and directing attention to the needs of clients as legitimate demands, advocates can confer symbolic capital on those to whom it is otherwise unavailable [57]. Though participants in the studies do not explain it in terms of symbolic capital, this role is apparent in the statements that appropriate funding is unattainable without the assistance of a good advocate (e.g. Mavromas [49]).

As Hastings and Matthews [5] argue, social service systems can be thought of as the ‘field of struggle’ in the Bourdieusian sense. Here, fields are different systems which are characterised by struggles – where ‘regularised, institutionalised unequal positions of social agents’ play out in competitive relations [5]. The field of struggles exists horizontally – cutting across all fields [25]. Success in the ‘field of struggle’ depends upon the ‘fit’ between habitus and the field. The ability to navigate, or play, in the field of struggles depends on one’s capital.

With regard to personsalisation, as a whole we can see a better alignment between middle-class capital and the service system. In part this stems from a greater amount of economic capital, however social and cultural capital play a key role, and indeed economic capital can also be converted into other forms of capital [25]. By passing greater choice and control to participants in personalisation schemes, these participants are also being passed the administrative and decision-making burdens that come with the empowerment [17]. Personalisation places autonomy of choice and decision making on to the participant, both allowing for autonomous and active decision making around care and often increasing the administrative burden of locating, coordinating, assessing and paying for care on to the person receiving care and/or their advocate, whether funded or familial [17]. This empowerment and encumbrance are two sides of the one coin within personalisation programs.

The very principles that underpin personalisation schemes mean that the programs are vulnerable to being designed in ways that privilege users who have the best capacity to navigate the system; those with the greatest capital. This is likely to be exacerbated by broader normative forces at play in the field of social services. Many welfare states in which personalisation has been adopted are characterised by residualist tendencies, whereby ‘welfare’ is positioned as a last resort after personal and family resources have become exhausted [11]. As a result, welfare supports are targeted at the poor [11]. Research on the sociology of social problems has described how broad social problems become framed as individual-level deficiencies [58]. Here, when particular groups fail to benefit from social programs those groups – and their behaviours – equally become the target of interventions and blamed for their lack of uptake. In the case of personalisation, over time this would see the focus of interventions shift to individuals seen as not making the ‘right choice’ about their care [2, 58]. In doing so, citizens are conceptualised as actor-consumers that are themselves failing to take to the system.

To deliver on their aims of more tailored care for all, personalisation schemes need to avoid this recursive policy trap. Attention also needs to be given to the design and implementation of such schemes in order to identify ways in which to overcome the inherent privileging of those with greater capital or that share institutional doxa [52]. For example, having well-funded advocacy programs can help participants to secure the right level of funding [49], while well-resourced and good quality care coordinator roles could help participants to identify and organise services that best meet their needs. Further, personalisation schemes must integrate the understanding that they have great potential to privilege middle class users, ideally from design stages but at least from now onwards. Personalisation programs must invest in monitoring and evaluation of uptake and advantage given to participants based on demographic features at least according to economic capital and potentially community interconnectedness and remoteness of living situations.

## Conclusion

Particularist approaches to social policy are supported on the basis that they enhance the choice and control of citizens, which in turn is expected to lead to better outcomes as well as empowering service users. This can be seen in personalisation policies, which are premised on enabling groups to exercise choice and control through the use of budgets or voucher systems whereby citizens can ‘purchase’ services that meet their needs. However, this rhetoric ignores differences in the distribution of choice and control across the population. While much research exploring differential outcomes of different socio-economic groups focuses on characteristics of individuals (and therefore explicitly or implicitly positions these individuals as deficient), recent work has argued for an approach that questions the systems through which these services are delivered [5, 6]. These authors argue that service systems are designed from a middle class ‘doxa’, thereby privileging middle class norms. That is, such systems favour skills and resources more commonly found in the middle class. Schemes based on personalisation require an unprecedented level of skills and resources at the individual level – requiring citizens to manage budgets and navigate hugely complex administrative systems. As a result, they are even more likely to result in the type of socioeconomic gradient that has been found in social and health service research.

To date, no research has sought to empirically study the link between socio-economic status and outcomes from personalisation schemes. In our review we identified a range of proxies for socio-economic position, which appear to be linked to the ability to navigate personalisation schemes and, in turn, achieve good outcomes. These factors can be clustered into Bourdieu’s three types of capital, which underpin class. Based on this analysis we argue there is good reason to suspect that personalisation schemes are entrenching social inequity. This is particularly concerning given growing international interest in these schemes, particularly for service users who already experience a range of inequities (e.g. people with the disability or the elderly).

## Data Availability

No primary data was collected for this study.
